# Changes in clinical parameters following adjunctive local sodium hypochlorite gel in minimally invasive nonsurgical therapy (MINST) of periodontal pockets: a 6-month randomized controlled clinical trial

**DOI:** 10.1007/s00784-021-03841-8

**Published:** 2021-03-09

**Authors:** Vincenzo Iorio-Siciliano, Luca Ramaglia, Gaetano Isola, Andrea Blasi, Giovanni E. Salvi, Anton Sculean

**Affiliations:** 1 Department of Periodontology, School of Dental Medicine, University of Naples Federico II, Via S. Pansini 5, 80131 Naples, Italy; 2 Department of Biomedical, Odontostomatological Sciences and of Morphological and Functional Immages, School of Dentistry, University of Messina, AOU Policlinico “G.Martino”, Via C.Valeria 1, 98125 Messina, Italy; 3 Department of General Surgery and Surgical-Medical Specialities, School of Dentistry University of Catania, Via Sofia 78, 95125 Catania, Italy; 4 Department of Periodontology, School of Dental Medicine, University of Bern, Freiburgstrasse 7, CH-3010 Bern, Switzerland

**Keywords:** Periodontitis, Periodontal pockets, Hypochlorite, Biofilm, Bleeding on probing, Nonsurgical periodontal debridement

## Abstract

**Background:**

The mechanical disruption and removal of the subgingival biofilm represent the most important step in the treatment of periodontitis. However, in deep periodontal pockets, mechanical removal of the subgingival biofilm is difficult and frequently incomplete. Preliminary findings indicate that the use of amino acid buffered sodium hypochlorite (NaOCl) gel may chemically destroy the bacterial biofilm and facilitate its mechanical removal.

**Objectives:**

To clinically evaluate the efficacy of minimally invasive nonsurgical therapy (MINST) of periodontal pockets with or without local application of an amino acid buffered sodium hypochlorite (NaOCl) gel.

**Materials and methods:**

Forty untreated patients diagnosed with severe/advanced periodontitis (i.e. stage III/IV) with a slow/moderate rate of progression (i.e. grade A/B) were randomly allocated in two treatment groups. In the test group, the periodontal pockets were treated by means of MINST and NaOCl gel application, while in the control group, treatment consisted of MINST alone. Full-mouth plaque scores (FMPS), full-mouth bleeding scores (FMBS), probing depths (PD), clinical attachment levels (CAL) and gingival recessions (GR) were assessed at baseline and at 6 months following therapy. The primary outcome variable was PD reduction at sites with PD ≥ 5 mm at baseline.

**Results:**

At 6 months, statistically significant differences between the two groups were found (*p* = 0.001) in terms of PD and CAL change. No statistically significant differences were found in terms of GR (*p* = 0.81). The number of sites with PD ≥ 5 mm and BOP (+) decreased statistically significantly (*p* = 0.001), i.e. from 85.3 to 2.2% in the test group and from 81.6 to 7.3% in the control group, respectively. Statistically significant differences between test and control groups were recorded at 6 months (*p* = 0.001). MINST + NaOCl compared to MINST alone decreased statistically significantly (*p* = 0.001) the probability of residual PDs ≥ 5 mm with BOP− (14.5% vs 18.3%) and BOP+ (2.2% vs. 7.2%).

**Conclusions:**

Within their limits, the present results indicate that (a) the use of MINST may represent a clinically valuable approach for nonsurgical therapy and (b) the application of NaOCl gel in conjunction with MINST may additionally improve the clinical outcomes compared to the use of MINST alone.

**Clinical relevance:**

In patients with untreated periodontitis, treatment of deep pockets by means of MINST in conjunction with a NaOCl gel may represent a valuable approach to additionally improve the clinical outcomes obtained with MINST alone

**Supplementary Information:**

The online version contains supplementary material available at 10.1007/s00784-021-03841-8.

## Introduction

The development and progression of periodontitis depend on the presence of pathogenic microorganisms organized in a supra/subgingival biofilm attached to the dental surface [1, 2]. The main goal of nonsurgical periodontal therapy is to eliminate the periodontal pathogenic biofilm from the tooth surfaces and from the periodontal pockets to reduce probing pocket depths and inflammation (i.e. bleeding on probing), ultimately arresting periodontal disease progression [2, 3]. Today, it is generally accepted that mechanical disruption and removal of the subgingival biofilm using hand and ultrasonic/sonic instruments represent the most important step in the treatment of periodontitis leading, in the great majority of cases, to successful clinical outcomes [2–4]. However, in certain clinical situations, such as the presence of deep periodontal pockets or deep furcation involvements, mechanical removal of the subgingival biofilm is difficult and frequently incomplete [5].

In the last years, the use of mini- and micro-instruments in combination with magnification loupes was suggested to more accurately eliminate the biofilm from deep periodontal pockets [6–8]. Clinical, microbiological and histologic findings appear to indicate that minimally invasive nonsurgical periodontal therapy may be a valuable option for the treatment of deep periodontal pockets [6–8].

Additionally, in the last decades, a number of novel strategies encompassing the use of locally delivered antiseptic and/or anti-inflammatory agents, antibiotics or photodynamic therapy, have been tested to enable a more accurate disruption and removal of the subgingival biofilm and to additionally improve the clinical outcomes and reduce the need for surgery [2, 9–11].

NaOCl has been suggested as a potential agent for the treatment of gingivitis [12] and, later, in the form of irrigation combined with mechanical debridement for the treatment of periodontitis [13].

Recently, a novel formulation consisting of NaOCl 0.95% and amino acids (glutamic acid, leucine, lysine) gel has been introduced to detoxify the root surfaces, to soften the calculus thus facilitating its removal by means of root planing [14, 15].

Findings from an “in vitro” study have shown that this novel NaOCl formulation acts have an antimicrobial effect, in particular against Gram-negative species associated with periodontitis, thus pointing to its potential use as an adjunctive topical antimicrobial in the treatment of periodontitis [14]. Subsequent findings from “in vitro” studies have shown that the application of the amino acid buffered hypochlorite solution had a positive effect on the survival, attachment and spreading of periodontal ligament cells onto root surfaces [15].

However, at present, the data on the potential clinical relevance of a local application of NaOCl used in conjunction with subgingival mechanical instrumentation is still limited [13, 16].

More recently, a novel protocol termed minimally invasive nonsurgical therapy (MINST) has been proposed for the treatment of isolated deep pockets associated with intrabony defects [17, 18]. Treatment of deep periodontal pockets by means of MINST consists of careful scaling and root planing using ultrasonic devices with delicate tips, mini-curettes and operating microscope under local anaesthesia [17, 18]. In a first study, the authors have treated intrabony periodontal defects with either MINST or minimally invasive surgical technique (MIST) [17]. The results at 3 and 6 months have failed to show any differences in terms of the clinical outcomes between the 2 procedures, thus suggesting that MINST may represent a valuable alternative to a surgical approach. An important observation was also the fact that treatment with MINST has led to an additional reduction of treatment chair time compared to MIST. A follow-up evaluation of the same patient population, together with findings made by other groups, has provided additional evidence suggesting that MINST may represent a valuable modality to successfully treat deep periodontal pockets associated with intrabony defects [18–20].

However, at present, according to the best of our knowledge, no data from randomized, controlled clinical studies are available evaluating the efficacy of MINST used with or without local application of an amino acid buffered sodium hypochlorite (NaOCl) gel in patients with untreated periodontitis.

Hence, the aim of the present randomized controlled clinical study was to evaluate the efficacy of minimally invasive nonsurgical debridement (MINST) of periodontal pockets with or without adjunct of amino acid buffered sodium hypochlorite (NaOCl) gel application over a period of 6 months.

## Materials and methods

### Study design

The study was designed as a double-arm, randomized controlled, superiority clinical trial. All periodontal pockets exhibiting probing depths (PD) of ≥ 5 mm were treated by means of MINST either alone (i.e. control group) or in combination with NaOCl gel application (i.e. test group). The study was conducted from May 2018 until December 2019. The study protocol was approved by the Commission on Research Ethics of the University of Messina (approval N°16/18).

Written informed consent was obtained from subjects and the study was conducted according to the Principles of the Declaration of Helsinki on experimentation involving human subjects. The research protocol was registered on Clinicaltrials.gov registry (registration number NCT04399187). The present trial was conducted according to the CONSORT statement (http://www.consort-statement.org). The null hypothesis of no statistically significant differences in terms of PD reduction between test and control procedure for the treatment of periodontal pockets was tested.

### Participants

All subjects enrolled in the study were recruited from the School of Dentistry, University of Messina, Italy. Data were collected in the same research center and then the statistical analysis was performed in the Department of Periodontology, University of Naples Federico II, Italy.

#### Eligibility criteria for participants

Inclusion criteria:

- Untreated patients diagnosed with severe/advanced periodontitis (i.e. stage III/IV) with slow/moderate rate of progression (i.e. grade A/B) [21]
- Age ≥ 18 years old;
- Patients with at least 10 teeth per arch;
- Presence at least of two teeth with PD ≥ 5 mm per quadrant;
- Single-rooted teeth or multi-rooted teeth without furcation involvement;

Exclusion criteria:

- Patients with systemic diseases;
- Pregnant or lactating;
- Tobacco smokers (> 10 cigarettes/day);
- Previous periodontal treatment in the last 2 years;
- Prolonged antibiotic treatment or anti-inflammatory treatment within 6 months prior to periodontal therapy;
- Furcation involvement;
- Acute periodontal or endodontic abscesses;
- Third molars

### Interventions

#### Clinical procedure

In the first session, all patients received a full-mouth supragingival scaling in order to remove the supragingival biofilm and calculus in combination with oral hygiene instructions and motivation.

**Fig. 1:**
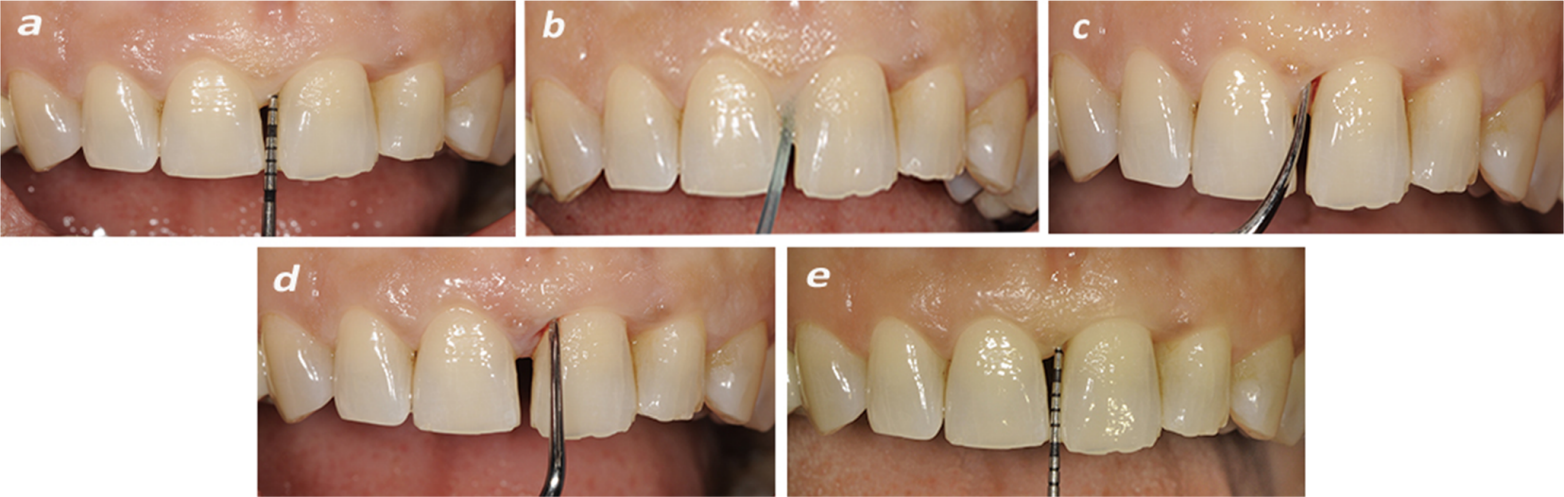
**a** A probing depth (PD) of 7 mm was recorded at baseline. **b** Prior to mechanical instrumentation the NaOCl gel was applied in the periodontal pocket for 30 s. **c** Subgingival debridement was performed using an ultrasonic scaler with a thin tip. **d** A gently root planning was made by means of Gracey micro-curette. **e** A probing depth of 3 mm was recorded at 6 months post-therapy

After 1 week all clinical parameters were recorded (Fig. 1a) and subjects were randomly assigned to the test or control procedures. The test group was treated as follows:

1. After local anaesthesia, an amino acid–buffered sodium hypochlorite gel (Perisolv®, Regedent AG, Zurich, Switzerland) was applied for 30 s in periodontal pockets with PD ³5 mm using a sterile syringe with a plastic needle. The tip was carefully inserted into the pocket until resistance was reached and was followed by its slow ejection (Fig. 1b). No rinsing was performed after the application of the gel.
2. MINST was performed by means of careful subgingival debridement using ultrasonic scalers with specific thin tips (Instrument PS®EMS Electro Medical System S.A., Nyon, Switzerland) (Fig. 1c) and Gracey micro-curettes (Hu-Friedy®, Chicago, IL, USA) in order to minimize the trauma for the soft tissues (Fig. 1d).
3. Application of amino acid buffered sodium hypochlorite gel and MINST was performed according to the manufacturer’s instructions.

**Fig. 2:**
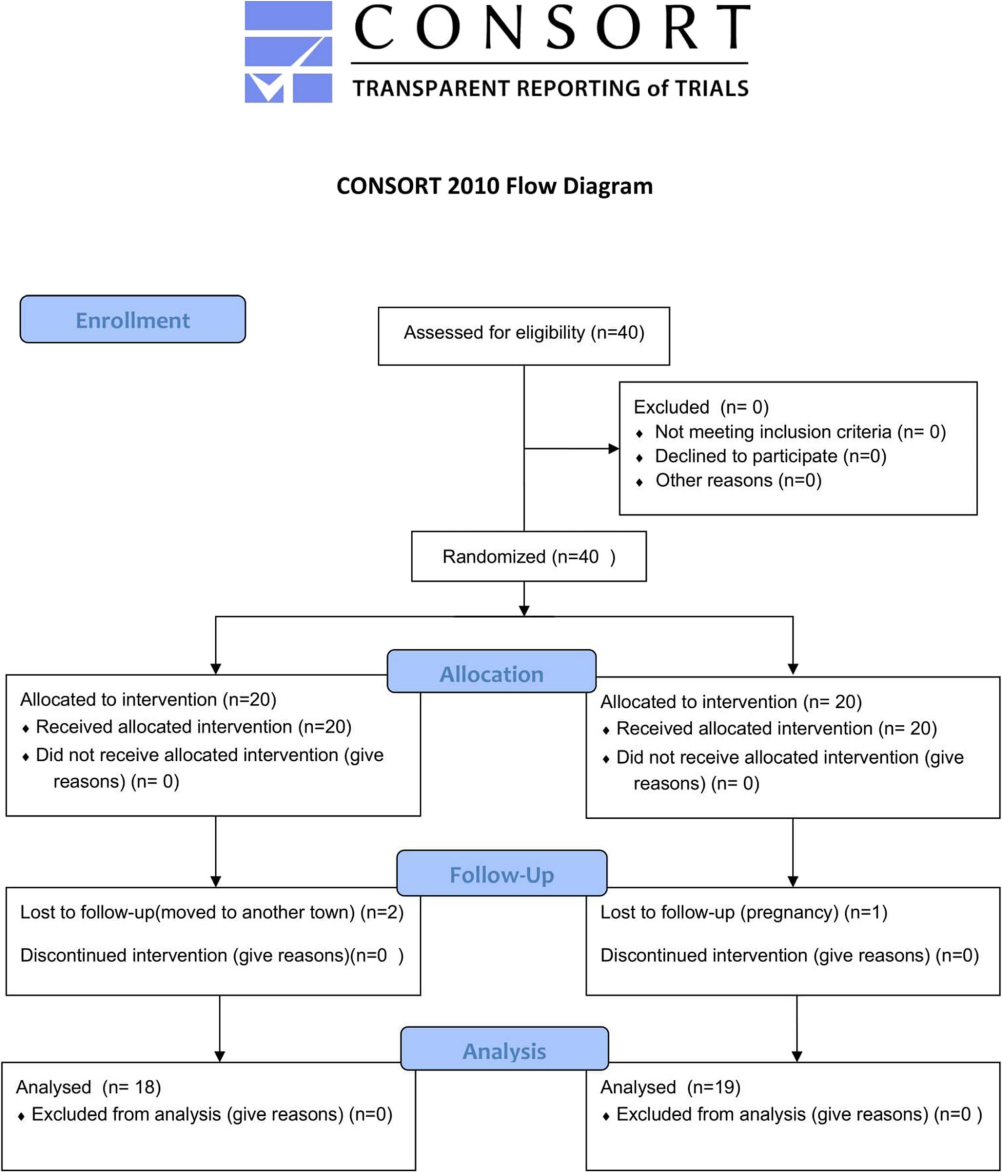
CONSORT flowchart

In the control group, treatment consisted of MINST alone without gel application.

All treatments were performed using × 4.0 magnification loupes (Univet®, Italy). At the end of the subgingival treatment, in both groups, full-mouth supragingival cleaning by means of a rubber cup and a polishing paste was performed. Patients were instructed to rinse twice daily with 0.12% chlorhexidine digluconate (Curasept ADS® Curaden AG, Kriens, Switzerland) for the first 2 weeks. No antibiotics were prescribed. Patients were recalled on a monthly basis for professional supragingival tooth cleaning and motivation during the entire study period of 6 months when the final evaluation was made.

### Outcome measures

The primary outcome variable was the probing depth (PD) reduction, defined as the distance from the gingival margin to the bottom of the pocket.

The secondary outcome variables were full-mouth plaque score (FMPS): percentage of tooth sites revealing the presence of plaque [22]; full-mouth bleeding score (FMBS): percentage of sites with bleeding on probing (BOP) [23]; clinical attachment level (CAL): distance from the cement-enamel junction (CEJ) to the bottom of the pocket and gingival recession (GR): distance from the gingival margin to the CEJ.

All clinical parameters were recorded at 6 sites per tooth by means of a manual periodontal probe (PCP-UNC 15®, Hu-Friedy, Chicago, IL, USA), applying a probing force of 0.2 N. All variables were recorded at baseline and after 6 months (Fig. 1e).

### Sample size calculation

The present study was designed to test a continuous response variable (i.e. PD) from independent control and experimental subjects with 1 control per experimental subject. In a previous study, using a similar design [24], the response within each subject group was normally distributed with a standard deviation of 0.7 mm. If the true difference in the means of the experimental and control group is 0.9 mm, a sample of 22 patients (11 patients per group) is needed to reject the null hypothesis that the population means of the experimental and control groups are equal with probability (power) 0.8. The type I error probability associated with this test of this null hypothesis is 0.05. In order to compensate for patients’ dropouts during the study period, a total of 40 subjects (i.e. 20 test and 20 control subjects) were enrolled in the study.

### Randomization

A computerized random number generator was used in order to random assign the subjects to experimental or control procedures. A simple randomization without restrictions was done. The allocation concealment was made associating even numbers to the test procedure and odd number to the control procedure. The cards with numbers were closed in opaque envelopes and treatment allocation was performed at the time of minimally invasive nonsurgical treatment by opening the envelope containing the number.

The random allocation sequence was generated by A.B., while participants were enrolled by I.G. in the School of Dentistry, University of Messina, Italy.

### Blinding and calibration

All patients enrolled in the study received periodontal therapy by the same periodontist (VIS). All parameters were recorded at baseline and after 6 months by 2 calibrated and masked examiners (I.G. and A.B.). Examiners attended a single training and calibration session on a total of 20 patients (kappa coefficient = 0.81). The calibration of all parameters was made in the same visit. The calibration meeting was performed at the School of Dentistry, University of Messina, Italy. Patients were not masked in respect to test and control procedures.

### Statistical analysis

The data analysis was performed using a commercially available statistical software (NCSS-PASS, NCSS, Kaysville, UT). The patient was considered as the statistical unit; however, an additional site-based analysis was also performed. All variables were expressed in millimetres with the exception of the FMPS and FMBS, which were reported in percentage.

Descriptive statistics (e.g. mean and standard deviation) were used to present the variables (e.g. FMPS, FMBS, PD, CAL and GR). For the statistical analysis, sites with PD ≥ 5 mm at baseline were considered. An unpaired *t*-test was applied to compare the mean age of participant at baseline. A chi-square test was used to compare gender and number of smokers. In addition, also the number and percentages of sites with PD ≥ 5 with BOP positive at baseline and after the 6-month follow-up period were compared using a chi-square test.

In order to avoid pseudo-replication, an average of data proceeding from the same patient was calculated and used for statistical analysis. An intra-group comparison was made with paired *t*-test between FMPS, FMBS, PD, CAL and GR values assessed at baseline and follow-up for both procedures (i.e. MINST + NaOCl gel and MINST alone). An inter-group comparison between test and control procedures was performed with an independent *t*-test for FMPS, FMBS, PD, CAL and GR at baseline, follow-up and for variations between baseline and follow-up values. In order to compare the frequency distribution of sites with residual PD between test and control groups, the Mantel-Haenszel *χ*^2^ test was used. In addition, a sub-analysis for distribution of treated teeth in each group (i.e. anterior *vs* posterior and maxillary teeth *vs* mandibular teeth) was performed by means of the Mantel-Haenszel *χ*^2^ test.

Cohen’s *D* was calculated to assess the effect size in mean differences between the treatment groups for changes in PD, CAL and GR.

A *p* value < 0.05 was set to accept a statistically significant difference.

## Results

### Participants and recruitment

Figure 2 illustrates the flow chart of the study. After screening, 40 patients fulfilling the inclusion criteria were recruited. At 6 months, a total of 3 patients were lost (dropouts). Two patients were lost in the test group (subjects moved to another town). In the control group, 1 patient was lost because she was pregnant. Therefore, a total of 37 patients (18 subjects for the test group and 19 for the control group) were available for the final examination (Fig. 2). The study was conducted from May 2018 till December 2019. No complications related to any of the two procedures were recorded. Patient recruitment and treatment started in May 2018 and was completed in December 2018. The last follow-up visit was completed in June 2019. Data analysis was performed in September 2019.

### Demographic characteristics

The characteristics of the patient population are presented in Table 1. Six males and 12 females (mean age 53.3 ± 9.8 years; range age 40–67 years) were included in the test group and 10 males and 9 females (48.5 ± 6.5 years; range age 36–63 years) were allocated to the control group. A total of 8 patients were smokers (< 10 cigarettes/day). No statistically significant differences (*p >* 0.05) were observed with respect to mean age, gender and smoking habits between the test and control group (Table 1).

**Table 1:** Patient population at baseline

	Test group (*N* = 18)	Control group (*N* = 19)	Significance (*p*)
Mean age (years)	53.3 ± 9.8	48.5 ± 6.5	0.43*
Range age (years)	40–67	36–63	
Gender (M/F)	6/12	10/9	0.19**
Smokers (*N*/%)	4; 22.2	4; 21.1	0.62**

*M*, male; *F*, female; *N*, number of patients

*Based on unpaired *t*-test

**Based on chi-square test

### Changes in FMPS and FMBS

Table 2 reports FMPS and FMBS at baseline and after 6-month follow-up. At baseline, FMPS was 47.1 ± 16.5% for the test group and 50.9 ± 12.4% for the control group, respectively. No statistically significant difference was found (*p* = 0.43) between groups. At a 6-month follow-up, a FMPS of 17.0 ± 4.8% and 17.6 ± 5.7% was recorded for the test and control group, respectively. No statistically significant differences were recorded (*p* = 0.72) between the test and control group. In both groups, a statistically significant change was found in terms of FMPS between baseline and 6-month follow-up (*p* = 0.001). At 6 months, a statistically significant improvement in mean FMBS was measured in both groups, i.e. from 39.8 ± 15.1 to 13.3 ± 6.0% in the test and from 43.8 ± 11.5 to 15.2 ± 6.0% in the control (*p* = 0.001) group, respectively. However, between the two groups, no statistically significant differences were found in terms of FMBS at baseline (*p* = 0.36) and at the 6-month follow-up (*p* = 0.35) (Table 2).

**Table 2:** Comparison of FMPS and FMBS at baseline and after 6-month follow-up

	Baseline	6 months	Significance (*p*)
FMPS (%)			
Test group	47.1 ± 16.5	17.0 ± 4.8	0.001**
Control group	50.9 ± 12.4	17.6 ± 5.7	0.001**
Significance (*p*)	0.43*	0.72*	
FMBS (%)			
Test group	39.8 ± 15.1	13.3 ± 6.0	0.001**
Control group	43.8 ± 11.5	15.2 ± 6.0	0.001**
Significance (*p*)	0.36*	0.35*	

*FMPS*, full-mouth plaque score; *FMBS*, full-mouth bleeding score

*Based on paired *t*-test

**Based on independent *t*-test

### Probing depth changes

After 6 months, PD decreased statistically significantly (*p* = 0.001) from 5.96 ± 1.07 to 3.46 ± 1.08 mm in the test group and from 6.01 ± 1.60 to 4.03 ± 1.74 mm in the control group, respectively. At baseline, no statistically significant differences between the two groups (5.96 ± 1.07 mm vs. 6.01 ± 1.60 mm) were noted (*p* = 0.50). At 6 months, a statistically significant difference (3.46 ± 1.08 mm vs. 4.03 ± 1.74 mm) was found, favouring the test group (*p* = 0.001). At 6 months, the comparison between the mean changes between the test group (2.49 ± 0.76 mm) and the control group (1.98 ± 0.80 mm) was statistically significant (*p* = 0.001) (Table 3). The effect size (Cohen’s *D*) of the PD changes from baseline to 6 months between two groups was *d* = 0.66 (CI 0.55–0.76).

**Table 3:** Comparison of probing depth (PD), clinical attachment level (CAL) and gingival recession (GR) at baseline and after the 6-month follow-up period

	Baseline	6 months	Changes	Significance (*p*)
PD (mm)				
Test group	5.96 ± 1.07	3.46 ± 1.08	2.49 ± 0.76	0.001**
Control group	6.01 ± 1.60	4.03 ± 1.74	1.98 ± 0.80	0.001**
Significance (*p*)	0.50*	0.001*	0.001*	
CAL (mm)				
Test group	6.24 ± 1.21	3.40 ± 2.16	2.84 ± 2.09	0.001**
Control group	6.41 ± 2.21	4.41 ± 3.02	2.01 ± 1.83	0.001**
Significance (*p*)	0.06*	0.001*	0.001*	
GR (mm)				
Test group	0.47 ± 1.22	0.78 ± 1.72	0.30 ± 1.16	0.81**
Control group	0.50 ± 1.33	0.76 ± 1.78	0.26 ± 0.97	0.81**
Significance (*p*)	0.73*	0.81*	0.42*	

*PD*, probing depth; *CAL*, clinical attachment level; *GR*, gingival recession

*Based on paired *t*-test

**Based on independent *t*-test

### Clinical attachment level changes

Six months after therapy, mean CAL changed from 6.24 ± 1.21 to 3.40 ± 2.16 mm in the test and from 6.41 ± 2.21 to 4.41 ± 3.02 mm in the control group, respectively. In both groups, a statistically significant difference was measured (*p* = 0.001). The inter-group comparison revealed a statistically not significant difference (*p* = 0.06) at baseline but yielded a statistically significant difference (*p* = 0.001) at 6 months (Table 3). The effect size (Cohen’s *D*) of the CAL changes from baseline to 6 months between two groups was *d* = 0.42 (CI 0.32–0.52).

### Gingival recession changes

The mean GR increased from 0.47±1.22 to 0.78 ± 1.72 mm in the test group and from 0.50 ± 1.33 to 0.76 ± 1.78 mm in the control group. However, the increase in GR from baseline to 6 months was not statistically significant in any of the 2 groups (*p* = 0.81). Furthermore, there were no statistically significant differences (*p* = 0.73) between the two groups at baseline and at 6 months (*p* = 0.81) (Table 3). The effect size (Cohen’s *D*) of the GR changes from baseline to 6 months between two groups was *d* = 0.04 (CI − 0.06–0.13).

### Number and percentages of sites with PD ≥ 5 mm with BOP positive

Table 4 summarized the number and percentages of sites with PD ≥ 5 mm with BOP. The number of sites with PD ≥ 5 mm and BOP decreased significantly (*p* = 0.001) from 763 (85.3%) to 20 (2.2%) for patients treated by means of MINST + NaOCl and from 594 (81.6%) to 53 (7.3%) for patients treated by means of MINST alone after 6-month follow-up. No statistically significant difference was recorded at baseline between the test and control group (*p* = 0.05). However, at 6 months, the differences between the two groups were statistically significant (*p* = 0.001) (Table 5).

**Table 4:** Number and percentages of sites with PD ≥ 5 with BOP positive at baseline and after the 6-month follow-up period

	Baseline	6 months	Significance (*p*)
Test groups	763/85.3	20/2.2	0.001*
Control groups	594/81.6	53/7.3	0.001*
Significance (*p*)	0.05*	0.001*	

*Based on the chi-square test

**Table 5:** Frequency distribution of sites with residual PD (*N*/%) with and without BOP positive after 6-month follow-up

	0–4 mm	5 mm	6 mm	7 mm	≥ 8 mm
Residual PD with BOP negative (*N*/%)					
Test group	665/74.3	86/9.6	44/4.9	0/0	0/0
Control group	496/68.1	91/12.5	28/3.8	8/1.0	8/1.0
Significance (*p*)	0.001*				
Residual PD with BOP positive (*N*/%)					
Test group	80/8.9	20/2.2	0/0	0/0	0/0
Control group	44/6.0	30/4.1	2/0.3	1/0.1	20/2.7
Significance (*p*)	0.001*				

*PD*, probing depth; *BOP*, bleeding on probing; *N*, number of sites

*Based on the Mantel-Haenszel *χ*^2^ test

### Frequency distribution of residual PD

Details of the frequency distributions of residual PD changes are illustrated in Table 5. Statistically significant differences were found in terms of residual PD without BOP and for BOP-positive sites in both groups (*p* = 0.001).

In the test group, 14.5% of sites displayed PD ≥ 5 mm without BOP, while the corresponding values were 18.3% in the control group. The percentage of sites with PD ≥ 5 mm with BOP amounted to 7.2% in patients treated by means of MINST alone with the corresponding value of 2.2% sites with PD = 5 mm with BOP positive in patients treated with MINST + NaOCl. No sites with PD > 5 mm and BOP positive were found in the test group (Table 5).

### Frequency distribution of sites with residual PD with BOP positive (*N*/%) after 6-month follow-up in respect to teeth location

A sub-analysis for the distribution of sites with residual PD with BOP positive is reported in Table 6. In anterior and posterior teeth, statistically significant differences were recorded comparing MINST + NaOCl and MINST alone (*p* = 0.001). Likewise, a statistically significant difference was found when in maxillary and mandibular sites test and control procedures were compared (*p* = 0.001) (Table 6).

**Table 6:** Frequency distribution of sites with residual PD with BOP positive (*N*/%) after 6-month follow-up in respect to teeth location

	0–4 mm	5 mm	6 mm	7 mm	≥ 8 mm
Residual PD with BOP positive (*N*/%)					
Anterior teeth					
Test group	0 (0)	0 (0)	0 (0)	0 (0)	0 (0)
Control group	8 (27.6)	5 (17.2)	1 (3.4)	0 (0)	15 (51.7)
Significance (*p*)	0.001*				
Posterior teeth					
Test group	80 (80.0)	20 (20.0)	0 (0)	0 (0)	0 (0)
Control group	36 (52.9)	25 (36.8)	1 (1.5)	1 (1.5)	5 (3.0)
Significance (*p*)	0.001*				
Maxillary teeth					
Test group	70 (77.8)	20 (22.2)	0 (0)	0 (0)	0 (0)
Control group	33 (47.1)	21 (30.0)	0 (0)	1 (1.4)	15 (21.4)
Significance (*p*)	0.001*				
Mandibular teeth					
Test group	10 (100.0)	0 (0)	0 (0)	0 (0)	0 (0)
Control group	11 (40.7)	9 (33.3)	2 (7.4)	0 (0)	5 (18.5)
Significance (*p*)	0.02*				

*PD*, probing depth; *BOP*, bleeding on probing; *N*, number of sites

*Based on the Mantel-Haenszel *χ*^2^ test

## Discussion

The present randomized controlled clinical trial has evaluated the outcomes obtained at 6 months by means of MINST with and without application of NaOCl in patients with untreated periodontitis exhibiting deep periodontal pockets. Both groups received exactly the same type of mechanical treatment (i.e. MINST), the only difference being the application of NaOCl in the test group prior to mechanical debridement. All pockets exhibiting probing depths (PD) of ≥ 4 mm were treated by MINST, but only pockets with PD ≥ 5 mm were considered for the statistical analysis.

At 6 months, PD decreased statistically significantly in the test group and control group, respectively. A closer analysis of the results revealed that the number of sites with PD ≥ 5 mm exhibiting BOP decreased statistically significantly in both groups, indicating excellent clinical outcomes. The obtained clinical outcomes can, on the one hand, be explained by the use of MINST consisting of careful subgingival debridement by means of ultrasonic scalers with specially designed thin tips and micro-curettes using high-magnification loupes. These findings are supported by results from previous studies, which have shown that MINST enables a thorough biofilm removal from the root surfaces and the periodontal pockets, reducing to a minimum the trauma of the soft tissues [17–20]. An important finding of previous studies was that at sites exhibiting intrabony defects, the use of MINST yielded similar outcomes to the surgical approach (i.e. MIST), thus pointing to the clinical relevance of this novel nonsurgical treatment modality as an alternative to the more invasive periodontal surgery [17–19].

On the other hand, it is important to be kept in mind that all the patients included in the study exhibited a high level of oral hygiene and received rigorous periodontal maintenance consisting of oral hygiene instructions and supragingival tooth cleaning performed on a monthly basis during the entire study period of 6 months.

These findings are in line with the results of a long-term study evaluating the outcomes of preventive dental treatment in a group of carefully monitored subjects who were motivated to maintain a high standard of oral hygiene and received regular supportive periodontal therapy. Today, there is ample evidence indicating that once probing depths are reduced and periodontal infection is controlled, the incidence of caries and periodontal disease as well as tooth mortality can be reduced to a minimum and kept stable over a long-time period (i.e. 30 years) [25].

An important aspect that needs to be discussed is that despite the fact that at 6 months after therapy, a dramatic reduction in the percentages of sites with PD ≥ 5 mm was measured in both groups; the magnitude of the improvement was statistically significantly higher when NaOCl gel was also applied. These clinical results appear to support the findings from “in vitro” studies which have provided evidence for the antibacterial effect of this novel NaOCl formulation and its positive effects on the survival, attachment and spreading of periodontal ligament cells [14, 15].

The present results are somewhat controversial to those very recently reported by Megally et al. [16]. In that study, a total of 365 sites in 32 patients enrolled in periodontal maintenance and exhibiting PD ≥ 5 mm were treated by means of repeated (i.e. at months 0, 4 and 8) subgingival debridement using ultrasonic tips, alone or with a NaOCl gel. However, at 12 months, the results have failed to reveal statistically significant differences between the 2 groups, suggesting no major advantages following the use of NaOCl gel. The discrepancy between our results and those reported by Megally et al. [16] can be explained by the use of a more accurate debridement approach (i.e. MINST) in conjunction with NaOCl in patients with untreated periodontitis. It has been repeatedly demonstrated that untreated periodontal pockets react more favourable to mechanical instrumentation compared to residual pockets in patients enrolled in maintenance [26]. Furthermore, it may also be speculated that deep pockets in patients with untreated periodontitis exhibit substantially higher amounts of biofilm and calculus, compared to patients with treated periodontitis and enrolled in maintenance. Conversely, in the present patient population, the use of NaOCl formulation might have had a higher potential to exert its antimicrobial and calculus softening properties, compared to those enrolled in the aforementioned study.

A limit of the present study can be the absence of radiographic analysis of treated sites. In a previous study [27], Nibali and co-workers reported a mean of radiographic bone level change of 2.93 mm at sites associated with intrabony defects treated by means MINST. In the present study, the radiographic evaluation was not performed because most parts of the sites with PD ≥ 5 mm were associated with supra-bony defects. In these defects, no or very limited bone gain can be expected after the treatment.

Since the healing capacity and immune response of each individual can significantly vary, the comparison of periodontal tissue response among different patients to the given clinical procedures could represent a limitation of the present study. This could be avoided by assigning test and control procedures within the same dentition. However, it would have been difficult to enrol sites with the same characteristics in terms of probing depth within the same dentition (i.e. sites with PD = 5 mm on the right side and sites with PD = 5 mm on the left side). For these reasons, the investigation was based on the patient and not on site.

Within their limits, the present results indicate that (a) the use of MINST may represent a clinically valuable approach for nonsurgical therapy and (b) the application of NaOCl gel in conjunction with MINST may additionally improve the clinical outcomes compared to the use of MINST alone.

## Supplementary Information

ESM 1 (DOC 219 kb)
